# CTDUNet: A Multimodal CNN–Transformer Dual U-Shaped Network with Coordinate Space Attention for *Camellia oleifera* Pests and Diseases Segmentation in Complex Environments

**DOI:** 10.3390/plants13162274

**Published:** 2024-08-15

**Authors:** Ruitian Guo, Ruopeng Zhang, Hao Zhou, Tunjun Xie, Yuting Peng, Xili Chen, Guo Yu, Fangying Wan, Lin Li, Yongzhong Zhang, Ruifeng Liu

**Affiliations:** 1 School of Electronic Information and Physics, Central South University of Forestry and Technology, Changsha 410004, China; 20211609@csuft.edu.cn (R.G.); ruopengzhang22@163.com (R.Z.); 20231100403@csuft.edu.cn (H.Z.); x18649644751@163.com (T.X.); 14789994693@163.com (Y.P.); 20212424@csuft.edu.cn (X.C.); t20010595@csuft.edu.cn (Y.Z.); 2 School of Business, Central South University of Forestry and Technology, Changsha 410004, China; 20214394@csuft.edu.cn; 3 School of Forestry, Central South University of Forestry and Technology, Changsha 410004, China; t20061093@csuft.edu.cn

**Keywords:** *Camellia oleifera*, multimodal, CTDUNet, Coordinate Space Attention, CNN–Transformer, segmentation, image and text integration, diseases and pests

## Abstract

*Camellia oleifera* is a crop of high economic value, yet it is particularly susceptible to various diseases and pests that significantly reduce its yield and quality. Consequently, the precise segmentation and classification of diseased Camellia leaves are vital for managing pests and diseases effectively. Deep learning exhibits significant advantages in the segmentation of plant diseases and pests, particularly in complex image processing and automated feature extraction. However, when employing single-modal models to segment *Camellia oleifera* diseases, three critical challenges arise: (A) lesions may closely resemble the colors of the complex background; (B) small sections of diseased leaves overlap; (C) the presence of multiple diseases on a single leaf. These factors considerably hinder segmentation accuracy. A novel multimodal model, CNN–Transformer Dual U-shaped Network (CTDUNet), based on a CNN–Transformer architecture, has been proposed to integrate image and text information. This model first utilizes text data to address the shortcomings of single-modal image features, enhancing its ability to distinguish lesions from environmental characteristics, even under conditions where they closely resemble one another. Additionally, we introduce Coordinate Space Attention (CSA), which focuses on the positional relationships between targets, thereby improving the segmentation of overlapping leaf edges. Furthermore, cross-attention (CA) is employed to align image and text features effectively, preserving local information and enhancing the perception and differentiation of various diseases. The CTDUNet model was evaluated on a self-made multimodal dataset compared against several models, including DeeplabV3+, UNet, PSPNet, Segformer, HrNet, and Language meets Vision Transformer (LViT). The experimental results demonstrate that CTDUNet achieved an mean Intersection over Union (mIoU) of 86.14%, surpassing both multimodal models and the best single-modal model by 3.91% and 5.84%, respectively. Additionally, CTDUNet exhibits high balance in the multi-class segmentation of *Camellia oleifera* diseases and pests. These results indicate the successful application of fused image and text multimodal information in the segmentation of Camellia disease, achieving outstanding performance.

## 1. Introduction

The image segmentation of plants has become indispensable in the identification and treatment of diseases in modern agriculture. *Camellia oleifera* is a vital economic crop in Asia [1,2,3]. Precise classification and identification of its diseases and pests are essential for implementing effective prevention and control measures. However, the current method for segmenting pests and diseases in *Camellia oleifera* remains predominantly manual or semi-automatic, which hinders accurate lesion segmentation and classification. Recent studies have showcased the remarkable efficacy of deep learning in plant image segmentation and classification, achieving high accuracy in key economic crops such as tomatoes, grapes, and rice, among others [4,5]. Leveraging deep learning for the precise segmentation of *Camellia oleifera* lesions not only facilitates the assessment of infection severity for subsequent treatment but also plays a pivotal role in distinguishing between different disease types. The implementation of automated processing plays a crucial role in the development of targeted prevention and control measures, making a significant contribution to enhancing both the yield and quality of *Camellia oleifera* [6]. However, research in the field of *Camellia oleifera* pest and disease segmentation using deep learning is relatively scarce [7,8]. There are three pressing issues that need to be addressed: (A) the absence of publicly available datasets based on the natural environment for *Camellia oleifera* pests and diseases; (B) the limitations of single-modal image features in capturing high-level semantic understanding restrict further enhancements in model accuracy; (C) the simplicity of datasets, with a single type of disease and most images being directly captured on a white background after picking, consequently lacking complex real-world environments. These three issues severely hinder research and practical applications in *Camellia oleifera* pest and disease segmentation.

To address the aforementioned issues, a new dataset of pests and diseases affecting *Camellia oleifera* was built in this study. All images were captured within authentic plantation environments, meticulously preserving the intrinsic characteristics of the leaves and lesions. Currently, segmentation technology based on image information has become mature. The use of single encoder–decoder structures allows for effective extraction and compression of feature information. However, the extraction of image features is susceptible to influence by external environmental factors [9], and the richness of features in a single modality is insufficient, making detailed extraction challenging. Furthermore, single-modality models often perform poorly when handling multiscale targets [10], making it difficult to effectively segment both large and small targets simultaneously. Considering the limitations of image feature extraction and the performance bottleneck of single-modality segmentation models, it is more feasible to use complementary and easily labeled information to compensate for these deficiencies. Therefore, we have turned our attention to multimodal technology. This advanced approach, which integrates and interacts with various forms of data, has demonstrated unique advantages and broad application potential in fields such as biometrics, visual question answering (VQA), image–text retrieval, and text-to-image generation [11]. Especially in the field of semantic segmentation, multimodal technology has made breakthrough advancements in deep learning applications for crops such as tomatoes and rice [12,13]. By integrating information from different modalities, the model has expanded multidimensional features, significantly enhancing the accuracy and effectiveness of segmentation. The integration of text and images particularly demonstrates an inherent complementarity: images inherently capture the visual aspects such as appearance, shape, and color of objects, while text conveys semantic information encompassing object names, attributes, and contextual details [14].

Currently, research in the multimodal fusion of images and text is mainly centered around two aspects: neural network fusion and alignment of image–text. Neural network fusion necessitates effective information propagation between different input modalities, which is crucial for achieving deeper integration and understanding [15]. Image–text alignment must ensure consistency between the representations of images and text in the semantic space, enabling consistent representation of similar semantic content across different modalities [16]. Hence, the intricate network structures and alignment mechanisms will present formidable challenges.

**Figure 1:**
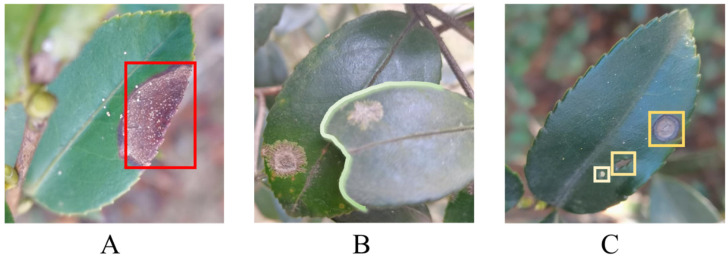
(**A**) The lesion, enclosed within a red box, is positioned at the leaf edge and shares a color resemblance with the background, complicating the accurate segmentation of its edge. (**B**) Two overlapping leaves, each exhibiting symptoms, pose a new challenge in their distinct identification. (**C**) A single leaf manifests three diseases in sequence from bottom to top in the yellow box: tea white spot, insect damage, and soft rot, demanding precise classification.

Semantic segmentation of an integrated image and text has demonstrated outstanding performance in medical image processing [17,18], where medical images are typically captured under fixed positions and angles, minimizing interference from external environments. In contrast, our self-made dataset of *Camellia oleifera* pests and diseases exhibits randomness in the number of leaves and types of symptoms. Moreover, the irregular arrangement of leaf positions and orientations poses significant challenges for accurately segmenting the lesions on Camellia oleifera. Our dataset, captured in natural environments, exhibits three distinct characteristics, as illustrated in Figure 1A. The natural environmental background colors are disorderly, with some colors closely resembling the lesions. In, Figure 1B, multiple overlapping leaves with lesions are present in a single image. In Figure 1C, individual leaves may exhibit multiple diseases simultaneously.

Medical image segmentation focuses on identifying and extracting affected areas, whereas the segmentation of *Camellia oleifera* pests and diseases requires the precise delineation of lesion regions, as well as accurate classification of these lesions. The inclusion of classification requirements undoubtedly introduces another significant challenge to the accuracy and complexity of pest and disease segmentation.

Upon reviewing the above, *Camellia oleifera* pest and disease segmentation presents three main challenges: (A) effectively integrating textual and visual features to enhance segmentation performance; (B) accurately identifying and extracting lesion areas based on complex backgrounds and conditions; (C) classifying multiple lesions accurately on the same leaf.

Image processing encompasses important techniques in computer vision, such as image classification [19], object detection, and instance segmentation [20]. Among these, semantic segmentation is particularly intriguing due to its ability to accurately label each pixel in an image, enabling a meticulous comprehension of the scene. In addition, researchers have expanded upon numerous image segmentation models. Among these, UNet has demonstrated outstanding performance in medical image segmentation [21]. The enhanced UNet++, which incorporates skip connections, alleviates the feature information gap between the encoder and decoder [22]. However, medical images typically only perform binary classification segmentation based on grayscale values, which fails to meet the complexity and diversity requirements of plant pest and disease datasets. To address these challenges in plant segmentation, Liu et al. [23] proposed PSOC-DRCNet, which utilizes Residual Adaptive Blocks (RABs) to detect various diseases in rice across diverse environments. Similarly, the Deeplab family enhances edge segmentation in complex environments for *Camellia oleifera* by introducing dilated convolutions to construct the ASPP module, effectively integrating multiscale information. Zhou et al. [24] improved the DeeplabV3+ model by enhancing the gated pyramid structure, effectively distinguishing similar features in *Camellia oleifera* pests and diseases. Chen et al. [6] introduced the Attention and Multi-Dimensional Feature Fusion Neural Network (AMDFNet), which accurately classifies *Camellia oleifera* pests and diseases. It is evident that deep learning has been extensively investigated in the context of *Camellia oleifera* pests and diseases. However, most of the aforementioned methodologies demonstrate limited efficacy in extracting features from high-quality images and exhibit sensitivity to the data quality and quantity, thereby constraining the model accuracy. Faced with the complexities present in our self-made dataset of Camellia, including lesions similar in color to the background, overlapping diseased leaves, and multiple lesions on the same leaf, conventional single-modal neural networks struggle to effectively address all three challenges simultaneously. Therefore, we are directing our focus towards multimodal technology.

Many research teams have successfully applied multimodal technology to the field of plant studies, achieving significant results. Hong et al. [25] utilized RGB and RGB-Depth (RGB-D) modal images for segmenting tomato fruits, aimed at production inspection and automated harvesting. Liu et al. [26] separately fed RGB and near-infrared (NIR) images into the ResNet50 backbone network, integrating features of different scales using a parallel attention mechanism, ultimately achieving segmentation of the main stem in tomatoes. However, both depth and near-infrared images require expensive mechanical equipment operated by professionals for acquisition. Considering the practicality and simplicity of the model, we are turning our focus towards straightforward text annotation. Thanks to the maturity of natural language processing (NLP) technology, there has been a proliferation of image segmentation efforts that use textual information to enhance the segmentation capabilities of models. In the medical field, Kim et al. [27] proposed the MONET model, which connects image and text to accurately annotate concepts in dermatological images. Multimodal tasks in VQA also integrate computer vision (CV) and NLP technologies [28]. In the domain of plant pathology, Zhou et al. [29] introduced the FHTW-Net framework, pioneering cross-modal retrieval for detecting rice leaf diseases, effectively addressing challenges in matching different image categories with complex textual descriptions. It is evident that the advancement of multimodal technology, which integrates images and text, has been remarkable; however, its application in pest and disease segmentation within the domain of *Camellia oleifera* remains limited. In this study, we propose a CNN–Transformer Dual U-shaped Network (CTDUNet) model that specifically focuses on integrating image and text for the accurate segmentation of pests and diseases.

The Contrastive Language-Image Pre-training (CLIP) model has pioneered large-scale vision–language pre-training, achieving significant performance improvements in multimodal tasks, particularly in matching and understanding images and text [30]. However, for extracting textual features specific to Camellia oleifera, which are typically brief and structured, BERT’s strengths are more advantageous. Bidirectional Encoder Representations from Transformers (BERT) focus on NLP, making it particularly adept at understanding and handling text data [31]. Therefore, we utilized BERT for processing the textual information related to *Camellia oleifera* pests and diseases. In handling natural language processing and computer vision tasks, Transformers have rapidly integrated into multimodal models due to their efficient feature representation and training capabilities. Among these models, Vision-and-Language Transformer (ViLT) is a multimodal model specifically designed for handling visual and language tasks. ViLT divides images into fixed-size patches and inputs them into the Transformer for feature fusion. However, this approach can result in the loss of some image details. Zhang et al. [32] further proposed MTGFE, which incorporates a multimodal fusion encoder using Transformer to combine image and text features at different levels, capturing richer cross-modal information. This spurred our interest in applying Transformer to semantic segmentation tasks. The Vision Transformer (ViT) cuts images into fixed-size patches to form input sequences and attaches positional encodings, thus demonstrating advantages in modeling global dependencies [33]. This approach lays the foundation for the model proposed in this paper, which combines CNNs with Transformers to process multimodal information.

However, the alignment and fusion of text and image features exhibit significant variations across different instances, necessitating further investigation in this domain. UNITER addresses this challenge by jointly encoding visual and textual features while employing a cross-modal attention mechanism to capture more nuanced correlations between images and text [34]. Gan et al. [35] proposed the Multimodal Fusion Network (MFN) with a Multi-Head Self-Attention mechanism to mitigate noise interference among different modalities. Additionally, Zhang et al. [36] introduced the Unified Adaptive Relevance Distinguishable Attention (UARDA) mechanism, which maximizes the distinction between relevant and irrelevant feature distributions for achieving superior semantic alignment. Similarly, in the field of image and text feature fusion, the Language meets Vision Transformer (LViT), proposed by Li et al. [18], has achieved remarkable success in medical image segmentation. Inspired by LViT, we extended their research to apply multimodal image–text fusion technology to the segmentation of plant diseases and pests.

In conclusion, to address the three primary challenges in the segmentation of *Camellia oleifera* diseases and pests, we have developed the CTDUNet model. The key innovations of our approach are summarized as follows:

(a) We have built a dataset of seven types of *Camellia oleifera* pests and diseases, consisting of 1400 image–text pairs. All images were captured directly from complex natural environments and annotated with textual information.

(b) We introduce a dual U-shaped visual–text model based on CNN–Transformer architecture (CTDUNet). This model deeply integrates Camellia images and text data, using text to compensate for image quality deficiencies and handle the complex environmental background of *Camellia oleifera* pests and diseases. The multimodal information significantly improves the segmentation and classification performance of CTDUNet.

(c) The innovative Coordinate Space Attention (CSA) mechanism was proposed as a lightweight attention module with long-range dependency capabilities. It effectively focuses on features across the C, H, and W dimensions, helping the model accurately locate important targets, concentrate on fine details, and effectively distinguish overlapping leaves.

(d) The experimental results show that CTDUNet achieves a mIoU of 86.14% on images with a resolution of only 224 × 224. This achieved a state-of-the-art performance among plant pest and disease segmentation models such as DeeplabV3+, UNet, PSPNet, HrNetV2, Segformer, and LViT, demonstrating the effectiveness of the network proposed in this paper.

In the following sections, we provide a detailed overview of our study. Section 2 covers the creation of the *Camellia oleifera* pest and disease image–text dataset, the experimental setup, and the model evaluation. Section 3 discusses the research findings and their significance. Section 4 explains the CTDUNet multimodal segmentation model, while Section 5 concludes with insights and future directions.

## 2. Results

### 2.1. Dataset

In the current field of *Camellia oleifera* pest and disease research, there is a lack of specialized image–text datasets. Therefore, we have constructed a multimodal dataset for *Camellia oleifera* pests and diseases. The dataset encompasses seven prevalent diseases, namely Tea White Scab, Worm Holes, Red Leaf Spot, Algae Leaf Spot, Tea Sooty Mold, Soft Rot, and Anthracnose.

**Figure 2:**
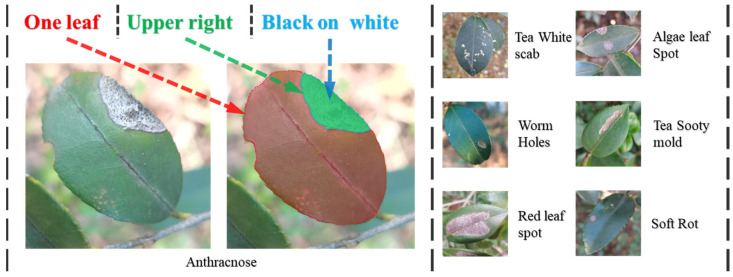
The text in the upper left corresponds to the labels of the image below, which indicate the number of leaves, the location of the lesions, and the color of the lesions. Below are the original and labeled images of Camellia anthracnose. To the right are six other Camellia diseases: Tea White Scab, Worm Holes, Red Leaf Spot, Algae Leaf Spot, Tea Sooty Mold, and Soft Rot.

This dataset primarily comprises text and images. As shown in Figure 2, for the text component, we employed manual annotation to ensure accuracy and consistency. The text descriptions are presented in concise phrases, covering three main elements: the number of leaves, the location of the lesions, and the color of the lesions. As shown in the diagram, the number of leaves is annotated with phrases such as “one leaf” or “two leaves”. The location of the lesions is described using a nine-grid division based on the center of the leaf, such as “middle and lower left”. The color of the lesions is accurately described based on actual observations, using terms like “red” or “white”. This method of description not only ensures simplicity but also enhances the portability and readability of the annotations.

For the image component, we used mobile phones as the shooting equipment, capturing images directly in their natural backgrounds. The images were collected from several locations: Yizhang County in Chenzhou, Hunan Province; You County, Hunan Province; Xingning, Guangdong Province; and Jiading Town in Xinfeng County, Jiangxi Province. To ensure the complexity and randomness of the data, we conducted the photo shoots during two time periods—December 2023 and March 2024—under various angles and lighting conditions. The images were captured with a 1:1 aspect ratio and a resolution of 3072 × 3072 pixels, resulting in a total of 2765 photos. To further enhance the model’s generalization ability and recognition accuracy, we preprocessed the image data. The preprocessing steps included scaling, blurring, and contrast adjustment, aiming to increase data diversity and robustness. Scaling alters the size of the input images, allowing the model to learn from variations in the object scale, which is critical for detecting objects at different distances or resolutions. Blurring introduces controlled noise, helping the model become more resilient to variations in the image quality or focus. Contrast adjustment modifies the brightness and color intensity, exposing the model to different lighting conditions and visual textures. Additionally, we compressed the image pixels to 224 × 224 to reduce the computational load and expedite model training. Ultimately, we retained 1400 photos and used Labelme to annotate the locations of *Camellia oleifera* diseases and generate mask images. We randomly selected 80% of the dataset (1120 images) for training and 20% (280 images) for validation, maintaining a consistent ratio for training and validation.

We collected several datasets on *Camellia oleifera* pests and diseases, though unfortunately, no public datasets are currently available. This underscores the innovative nature of our work, particularly since there are also no publicly available multimodal datasets for these pests and diseases. As illustrated in Table 1, the first dataset contains fewer disease categories and does not account for complex backgrounds. The second dataset addresses this issue but requires an extensive number of images. Both datasets focus solely on classification rather than segmentation, further emphasizing the pioneering aspect of our research.

### 2.2. Model Evaluation Indicator

We evaluate the effectiveness of the model using four metrics: mIoU, Dice, Precision, and Recall. Firstly, we define four types of regions: The True Positive (TP) category represents regions that exhibit actual diseases and are accurately predicted as diseased. Conversely, the True Negative (TN) category encompasses regions that are genuinely healthy and correctly identified as such. On the other hand, False Positive (FP) refers to regions that are actually diseased but erroneously classified as healthy. Lastly, False Negative (FN) pertains to regions that are truly healthy but mistakenly labeled as diseased.

Precision measures the proportion of correctly predicted diseased regions (TP) among all the predicted diseased regions (TP + FP). The expression is illustrated in Equation (1).

(1) $$Precision=\frac{TP}{TP+FP}$$

Recall indicates the proportion of correctly predicted diseased regions (TP) among all actual diseased regions (TP + FN). It reflects the model’s ability to identify positive samples correctly. The expression is represented by the following equation:

(2) $$Recall=\frac{TP}{TP+FN}$$

IoU (Intersection over Union) is used to measure the similarity between predicted and true diseased regions. ${IoU}_{i}$ for class i is calculated as the ratio of intersection to union of the predicted${\;A}_{i}\;$and $B_{i}\;$true regions. The expression is shown in Equation (3). mIoU measures the overlap between the predicted and ground truth segmentation masks across all the classes, making it ideal for evaluating the boundary delineation of *Camellia oleifera* pests and diseases. It provides a balanced assessment of the segmentation performance, considering both false positives and false negatives, thus enhancing the sensitivity to misclassifications. For these reasons, we chose mIoU as the primary metric for evaluating our model’s segmentation performance. The expression is further illustrated in Equation (4).

(3) $${IoU}_{i}=\frac{\left|A_{i}\cap B_{i}\right|}{\left|A_{i}{\cup B}_{i}\right|}$$

(4) $$mIoU=\frac{1}{N}\sum_{i=1}^{N}I{oU}_{i}$$

Dice (Dice coefficient) measures the similarity between predicted and true diseased regions. It is sensitive to class imbalance and effective for evaluating the segmentation of small targets. The expression is illustrated in Equation (5).

(5) $${Dice}_{i}=\frac{2\left|A_{i}\cap B_{i}\right|}{\left|A_{i}\right|+\left|B_{i}\right|}$$

### 2.3. Experiment Setup

The experiments in this study were conducted under a unified hardware and software environment to ensure efficient model training and effective comparison of the experiments. Table 2 presents the hardware configuration and environment setup used for training and validation. In our study, we applied grid search to optimize the hyperparameters of our network. We defined a range of values for each hyperparameter, such as learning rate, batch size, and regularization parameters. The grid search method then exhaustively tested each combination of these parameters. By evaluating the model’s performance for each combination, we identified the optimal set of hyperparameters that provided the best results in terms of segmentation accuracy. The selected hyperparameters, determined through this process, are listed in Table 3.

### 2.4. Analysis and Comparison of the Model Results

To evaluate the performance of our model and demonstrate the benefits of text annotations in multimodal technology, we conducted comparative experiments on our self-made camellia disease and pest dataset. Our model was compared to several state-of-the-art image segmentation models, including DeeplabV3+ [24], UNet [37], PSPNet [38], HrNetV2 [39], Segformer [40], and LViT.

For the mainstream segmentation models currently under study, we also experimented with different backbones to enhance the contrast. The segmentation performance metrics of all the models are detailed in Table 4. As shown, the PSPNet-MobilenetV2 model achieves a mIoU of only 61.73%, demonstrating the complexity of our self-made camellia disease and pest dataset. Traditional models struggle to meet high-precision segmentation requirements, finding it particularly challenging to address the three complex issues mentioned earlier. However, among the single-modal segmentation models, Segformer demonstrates outstanding performance, with Segformer-b5 achieving a mIoU of 82.21%. This superior performance can be attributed to Segformer’s Hierarchical Transformer, which effectively extracts image features at different scales [41]. This capability is particularly beneficial for detecting *Camellia oleifera* diseases and pests of different sizes and shapes, especially small targets in complex backgrounds. Despite this, its Dice coefficient performance is not as remarkable, indicating an imbalance in target recognition across all categories. The comparison also highlights the effectiveness of incorporating textual information in enhancing multi-class segmentation tasks. Our model demonstrates a 5.84% improvement in mIoU compared to LViT. This demonstrates that the proposed CSA can further enhance the capability of multimodal models to handle the challenges posed by complex backgrounds, thereby meeting the high demands of segmenting *Camellia oleifera* pests and diseases.

**Figure 3:**
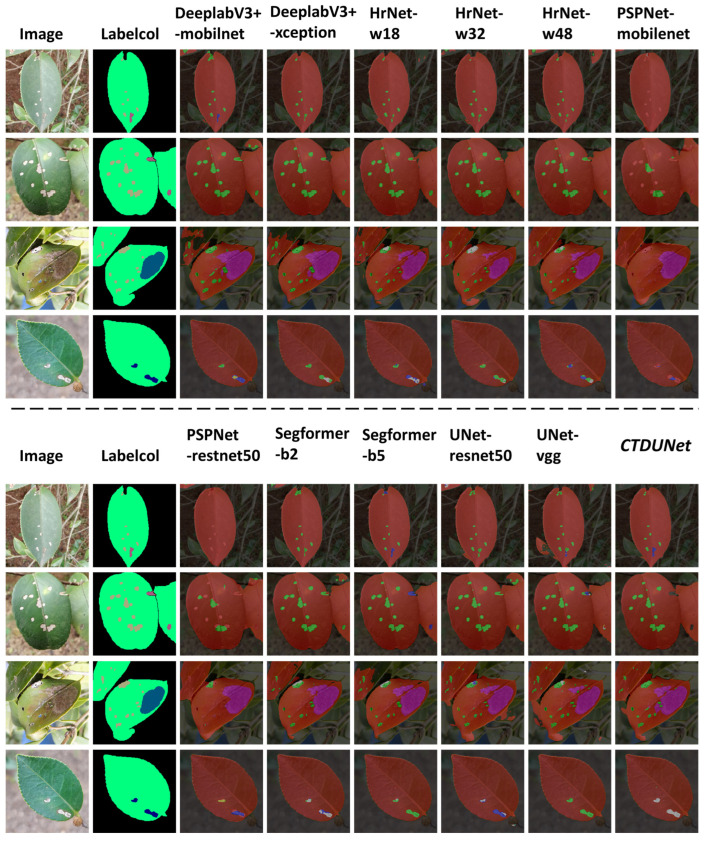
The visual differences in segmentation between the CTDUNet model and other models are demonstrated with four *Camellia oleifera* pest and disease images, including the aforementioned challenges.

The visualizations of all the models are shown in Figure 3. To provide a more intuitive comparison of the segmentation results, we overlaid the mask images onto the original images. In the overlay, red represents the leaves, green represents Tea White Scab disease, yellow represents Worm Holes disease, blue represents Tea Sooty Mold disease, pink represents Red Leaf Spot disease, cyan represents Soft Rot disease, gray represents Anthracnose disease, and dark red represents Algae Leaf Spot disease. 

The results clearly indicate that PSPNet-MobilenetV2 incorrectly identifies disease spots as leaves, leading to noticeable segmentation errors. This effectively explains why PSPNet-MobilenetV2’s performance metrics are lower. This issue may stem from the design of the MobileNetV2 backbone network, which limits the richness of deep feature extraction and thus fails to effectively distinguish similar regions. In contrast, our proposed model utilizes textual guidance to effectively integrate both textual and visual features, facilitating accurate differentiation between diseases affecting *Camellia oleifera* and environmental factors in first image. Furthermore, a notable distinction arises when confronted with overlapping leaves. As shown in the third image in Figure 3, the CTDUNet model proposed in this study can clearly delineate the boundaries between them rather than merging them into the same leaf or producing jagged edges, as compared to the visualization results of HrNetV2-W18. This demonstrates that the CSA mechanism proposed in this study, which extends to three dimensions of feature attention, can accurately preserve positional information. In our self-made dataset, another complex situation involves single leaves exhibiting multiple diseases simultaneously, as shown in the second picture in Figure 3. In the provided sample images, a leaf exhibits symptoms associated with two diseases: Tea Sooty Mold disease and Tea White Scab. They manifest as minuscule shapes with semblable colors, thereby posing challenges for precise segmentation and classification. DeeplabV3+-Xception failed to segment the Tea Sooty Mold lesions in the image, likely due to its ASPP module using dilated convolutions. While effective in expanding the receptive field, this approach also exacerbates the loss of fine details, particularly for small features [42]. On the other hand, UNet-Vgg misclassified Tea Sooty Mold as Tea White Scab in the same image. Its simplistic skip connections structure is insufficient to capture and differentiate subtle features among multiple categories, especially when the categories are numerous or exhibit significant similarities [43]. Our model clearly delineated the contours of the two types of lesions—Worm Holes and Tea White Scab—and correctly classified them. This demonstrates the superior performance of our model in multi-classification tasks and the effectiveness of the proposed modules.

### 2.5. Comparison of Coordinate Space Attention

The CTDUNet model is primarily structured with a dual U-shaped network consisting of CNN and Transformer architectures, where the CNN network employs Vgg16 as the backbone for image feature extraction. However, due to the complexity of the self-made *Camellia oleifera* dataset created in this study, it faces challenges in handling three specific issues.

Therefore, we proposed the Coordinate Space Attention (CSA) mechanism, which focuses on image features from three dimensions: C, H, and W. Leveraging its perceptual capabilities of global information, the CSA enhances the model’s ability to recognize and extract features of diseases similar to the environment and subtle diseases. To validate its effectiveness, we compared the performance of the CSA mechanism with other attention mechanisms, and the results are shown in Table 5. The introduction of CSA leads to a remarkable enhancement in the segmentation metrics of the model, surpassing other attention mechanisms by a significant margin. Specifically, Coordinate Attention performs lower than the CSA module in terms of mIoU. This highlights the limitations of dual-channel encoding, whereas CSA can encode and capture positional relationships in three dimensions. Compared to SE attention, CSA addresses the absence of positional information in feature representation, resulting in an improvement of the Dice metric from 89.26% to 92.45% Additionally, CSA overcomes the shortcomings of CBAM in extracting long-range relationships, leading to a significant overall performance enhancement.

### 2.6. Ablation Experiment

To address the aforementioned challenges in *Camellia oleifera* pest and disease segmentation, we proposed the CTDUNet model based on LViT [18], which represents a significant innovation in handling multimodal tasks. Our approach, based on a CNN–Transformer architecture with a dual U-shaped visual–textual model, aims to enhance the handling of fused data from Camellia images and text.

In the U-shaped CNN branch, the model employs Vgg16 as the backbone for comprehensive image feature extraction. To further enhance feature extraction, a CSA mechanism is integrated at the junction where sampled outputs from the first four layers connect with DownViT. This CSA mechanism focuses on features across the C, H, and W dimensions, ensuring the model captures more detailed information about Camellia, thus improving the segmentation accuracy. Furthermore, the last layer’s downsampling output from the backbone undergoes processing through the SPPF module, generating output feature maps that encapsulate multiscale information. The SPPF module achieves multiscale feature fusion by employing pooling operations at various scales, thereby augmenting the model’s capacity to discern objects across diverse scales.

In the U-shaped ViT branch of the model, text information is vectorized using BERT to convert it into high-dimensional feature representations. Initially, the cross-attention DownViT module (CA-DownViT) facilitates the fusion of textual features with image features, leveraging the complementary nature of both modalities. Subsequently, Transformer encoders process cross-modal information to further enhance feature expression and semantic understanding. The fused features are then propagated back to the CNN branch layer by layer and jointly input into the pixel-level attention module (PLAM), along with the image features output by the backbone. The module effectively refines the features at the pixel level, thereby enhancing the model’s capability to extract intricate details. Subsequently, by employing layer-wise upsampling, multiscale features are progressively integrated to achieve precise predictions on the input images.

**Figure 4:**
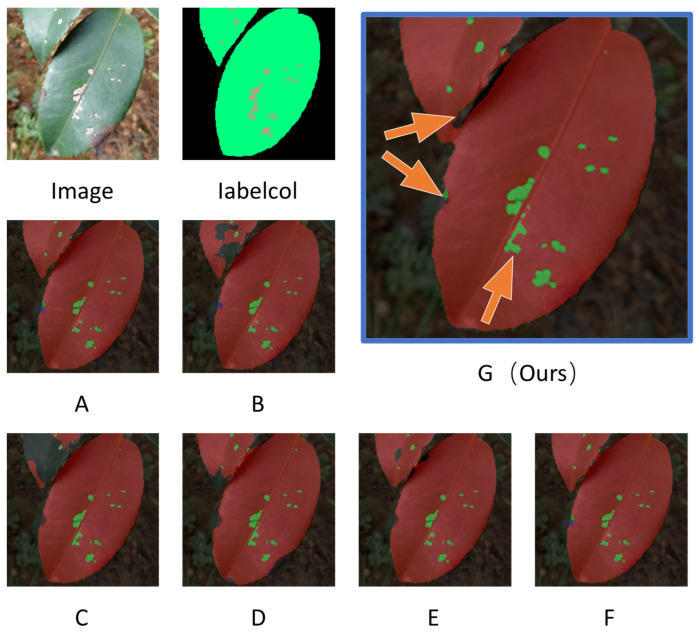
The entire figure represents the visualization process of the ablation experiments, where sections (**A**–**G**) correspond to the order listed from top to bottom in Table 6. We used arrows to indicate the areas in distinguishing boundaries and subtle features in the image, as shown in sections G.

To more intuitively demonstrate the performance improvements and effectiveness, we systematically added each module and compared their impact, observing changes in model performance. The performance metrics comparison is shown in Table 6, and the visual comparison is shown in Figure 4. In the visualized images, we focus primarily on the changes indicated by the three orange arrows. By incorporating the backbone into the base network, we observed a rapid 3.36% increase in the mIoU, indicating that deeper feature extraction significantly enhances the segmentation performance for the complex characteristics of *Camellia oleifera* pests and diseases. However, when we removed the text information, the model’s performance plummeted, even falling below that of the base network. Moreover, as shown in Figure 4C, there was a loss in the overall segmentation accuracy of the leaves. This highlights the critical role of text information in feature extraction. We further enhanced feature fusion using the SPPF and CA-DownViT modules, achieving modest improvements. The results showed that introducing the proposed CSA mechanism alone increased the Dice coefficient from 88.70% to 91.67%, demonstrating the module’s effectiveness in extracting positional information correlations and aiding in the differentiation of leaves, as shown in Figure 4E. Our CTDUNet model achieved Dice and mIoU scores of 92.45% and 86.14%, respectively. This validates the effectiveness of each module in improving the performance and addressing the three major challenges posed by the complex environments of *Camellia oleifera* pests and diseases. Finally, we tested the CTDUNet model without text information, resulting in a mIoU of only 80.73%. This further confirms the importance of text-guided image information for precise segmentation.

### 2.7. Label Balance

To further validate the effectiveness and balance of feature learning in CTDUNet, rather than focusing solely on the precise recognition of specific labels, we computed IoU scores across nine classes for seven models, as shown in Table 7. These include background, leaves, and seven types of *Camellia oleifera* diseases and pests. It can be observed that, for the background and leaves, which are larger target objects, models such as DeeplabV3+, PSPNet, and HrNetV2 show IoU differences of over 30% compared to smaller target disease categories. This indicates that these three models struggle to accurately segment *Camellia oleifera* diseases and pests, highlighting an imbalance in segmentation across the samples.

**Figure 5:**
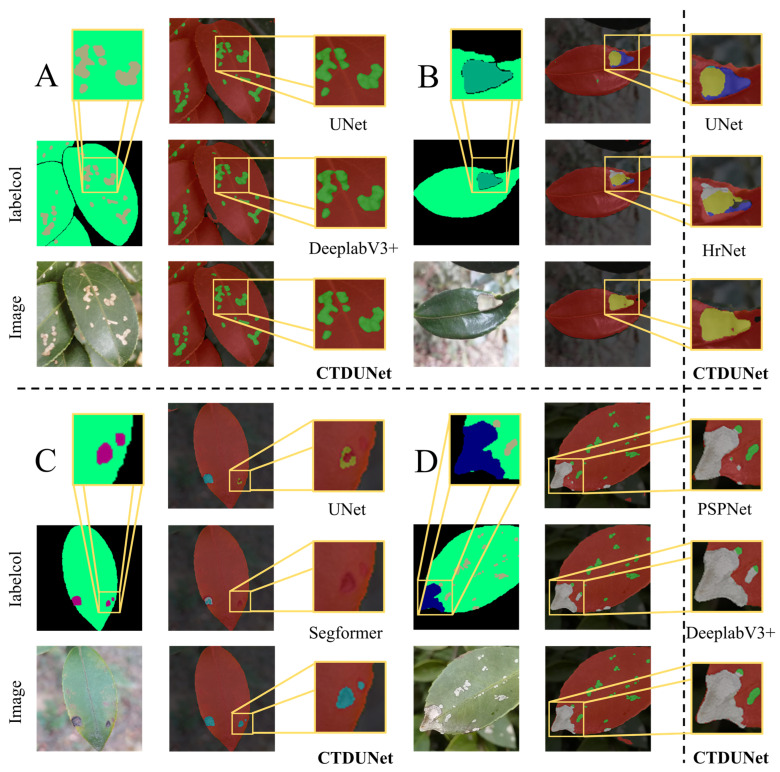
The detailed comparison of the CTDUNet model with other models clearly demonstrates CTDUNet’s balanced segmentation across different categories. Section **A**–**D** includes typical examples of *Camellia oleifera* pests and diseases that are challenging to segment, alongside a comparison of segmentation results from the CTDUNet model and other models.

The Tea White Scab’s features are notably small, making them prone to being overlooked in target recognition. As shown in Figure 5A, compared to the DeeplabV3+ and UNet models, CTDUNet excels in segmenting two discontinuous lesions, significantly enhancing the segmentation accuracy for small targets. The spots of Worm Holes and Soft Rot disease are relatively indistinct, with the colors closely resembling the background, posing challenges for differentiation. The visual results from the models also reveal that, when confronted with these three complex diseases and pests, most models exhibit segmentation errors. However, LViT and the CTDUNet model proposed in this paper demonstrate lower error rates and clearer segmentation edges, indicating the effectiveness of guiding image information with textual cues.

In the loss function of the model proposed in this paper, considering the disproportionately large proportions of background and leaves in the labels, a pixel-wise weight allocation function was employed during training to redistribute the proportions of each label in the loss function. This adjustment allows the model to focus more on small sample diseases. As shown in Figure 5B,C, the pixel-wise weight allocation function aids CTDUNet in accurately identifying disease types, effectively reducing segmentation errors. Additionally, in Figure 5D, our model successfully delineates the boundaries between two adjacent lesions, thereby enhancing its classification capability. The mIoU values for the aforementioned three disease categories are, respectively, 75.29%, 78.96%, and 94.67%, indicating that our proposed model can effectively adapt to complex multi-class segmentation tasks while ensuring balance among the segmentation categories.

## 3. Discussion

This research tackles the complexity of segmenting *Camellia oleifera* pests and diseases in real-world settings. In practice, isolating individual leaves and identifying them against a white background is impractical; meaningful segmentation must occur directly within the natural environment. In previous studies, researchers primarily relied on single-modal approaches, particularly image-based segmentation methods, to tackle the problem of plant disease detection. These methods focused solely on visual features extracted from images, often neglecting the rich semantic information that could be obtained from textual data. While these earlier models achieved moderate success, they struggled to accurately segment and classify diseases in complex environments, especially when faced with overlapping leaves or multiple diseases present on the same leaf. Our work builds upon single-modal segmentation methods, advancing them through the integration of a CNN–Transformer-based dual U-shaped visual–language multimodal model (CTDUNet). 

A pivotal element of our approach was the precise construction of a dataset comprising images and textual information on *Camellia oleifera* diseases. Unlike the existing datasets, our dataset retains the natural environment, capturing images without artificial intervention and thereby preserving the raw characteristics of the data to the greatest extent possible.

The dual U-shaped network structure of CTDUNet proved indispensable in addressing these challenges. Drawing from unimodal feature processing, the model employs two networks to extract features from each modality separately. The key innovation lies in the integration of image and textual information to process and accurately segment low-resolution images (224 × 224 pixels). This integration compensates for deficiencies in high-level semantic information typically present in image data alone, ensuring more precise segmentation despite limitations in the image quality. Compared to previous models, CTDUNet demonstrates superior performance, particularly in complex environmental settings, underscoring the effectiveness of textual information in guiding the segmentation process.

The CSA mechanism, which focuses on the C, H, and W dimensions, plays a crucial role in enhancing the model’s accuracy by enabling it to focus on the details of disease lesions. This capability is especially important when dealing with overlapping leaves or multiple diseases on the same leaf, situations that have traditionally been challenging for earlier models.

Furthermore, the cross-attention (CA) mechanism within our model ensures consistent alignment between textual and image features, leading to more accurate segmentation outcomes. The PLAM further enhances this alignment by merging channel features with similar semantics, preserving local image feature information that is critical for distinguishing between different disease symptoms.

We introduced a combined loss function that integrates Focal Loss and Dice Loss, addressing a significant challenge in segmentation tasks: class imbalance. This dual loss function not only enhances the model’s ability to focus on difficult-to-classify samples but also directly optimizes the segmentation metrics, resulting in a more robust performance. The pixel-wise weight allocation (PWWA) function refines this process further by optimizing the loss function for each diseased pixel type, thereby improving the model’s training effectiveness.

In the context of previous studies, which primarily relied on image-based methods with limited consideration of text-image interactions, our model’s ability to cross-fuse these modalities represents a substantial advancement. This suggests that integrating textual information can significantly enhance the segmentation process, a finding with potentially broad implications for the field of plant disease detection.

Nevertheless, our study has limitations. The model’s performance is heavily dependent on the quality and diversity of the annotated dataset. In cases where data are sparse or annotations are inconsistent, the model’s effectiveness may be compromised. Additionally, while our model performs well at low resolutions, further research is necessary to explore its scalability and application on higher-resolution images.

Future work will focus on introducing semi-supervised learning techniques guided by textual information. By leveraging the rich semantic content and contextual relationships encoded in text, we aim to enhance the model’s flexibility and adaptability further. This approach will enable more effective learning from limited labeled data, potentially overcoming some of the current limitations and paving the way for new methods in the segmentation and classification of plant diseases and pests across diverse and real-world environments.

## 4. Materials and Methods

In this study, we propose a dual U-shaped image–text model based on a CNN–Transformer architecture. The model utilizes Vgg16 [48] as the backbone in the U-shaped CNN branch to extract comprehensive image features. The CSA module is placed at the connection points of the first four sampling layers with DownViT, focusing on features from the C, H, and W dimensions. The SPPF module receives the output of the final downsampling layer from the backbone, generating an output feature map that encompasses multiscale information.

**Figure 6:**
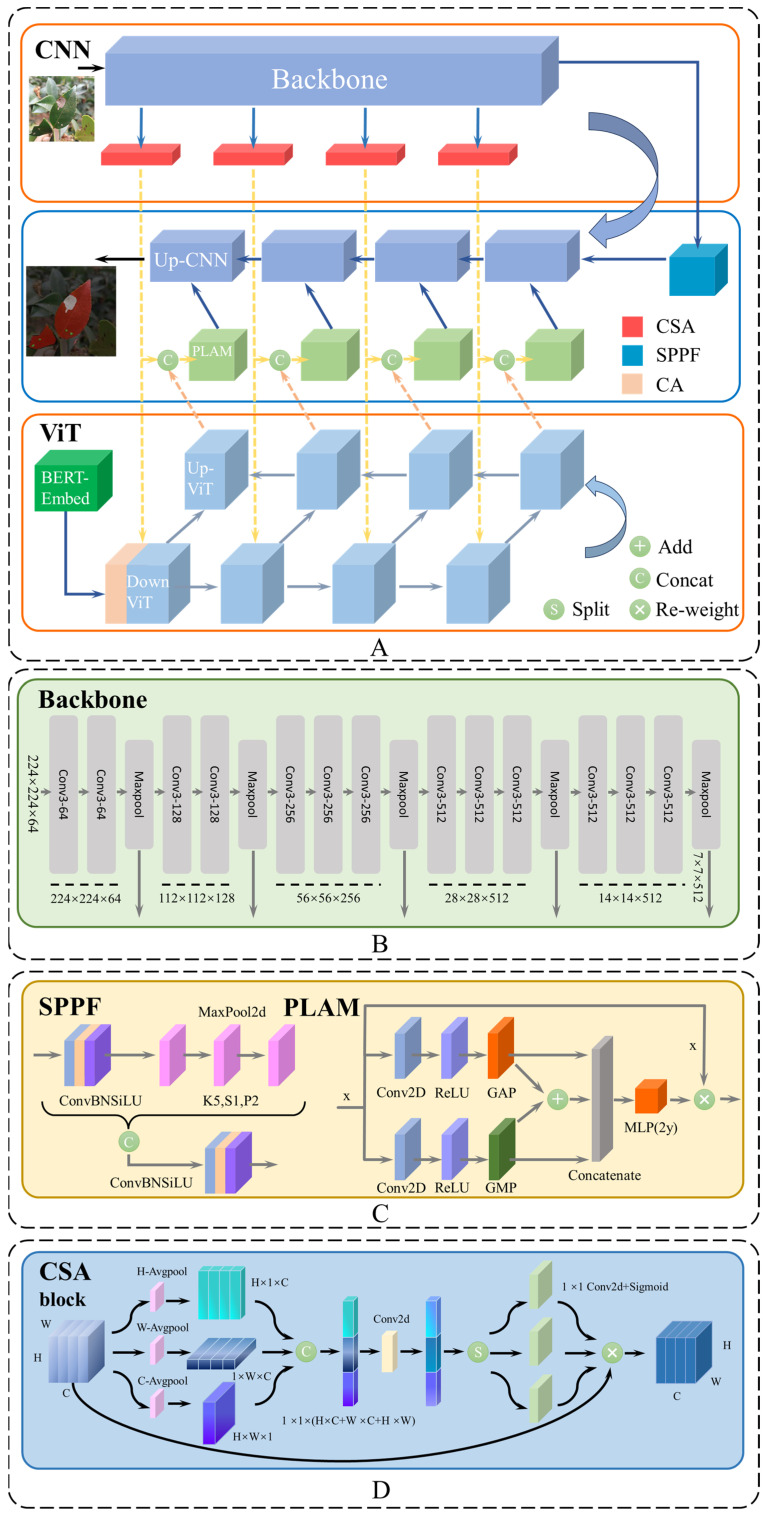
(**A**)The overall schematic of CTDUNet, a multimodal dual U-shaped architecture based on CNN–Transformer. (**B**) The network structure of the backbone within the CNN branch. (**C**) The network structures of SPPF and PLAM. (**D**)The network structure of the CSA block.

In the U-shaped ViT branch, BERT is used to vectorize the text. The CA-DownViT first performs cross-fusion of text and image features. Subsequently, a Transformer is employed to process cross-modal information. The fused features are progressively passed back to the CNN branch and, along with the backbone’s output image features, are fed into the PLAM to enhance the model’s ability to extract detailed features. Finally, multiscale feature fusion is achieved through iterative upsampling. The overall framework of the model is shown in Figure 6A.

### 4.1. CNN Branch

The U-shaped CNN branch primarily handles image information by image features and serves as the segmentation head to produce the final prediction results. We utilize the backbone to act as the downsampling branch of the U-shaped CNN, where the image undergoes successive convolution layers. Following this, MaxPool is employed to acquire downsampling feature maps at five different dimensional levels. The first two downsampling feature outputs undergo two layers of convolution, while the subsequent three undergo three layers of convolution. The specific process is illustrated in Equation (6).

(6) $$B_{i}=MaxPool\left({{Conv}_{i}\left(\cdot \right)}^{2/3}\right)$$

The first four downsampling feature maps are input into our proposed CSA mechanism, which attends to feature information across three dimensions and then optimizes the output features transmitted to DownViT. We employ SPPF to receive the last downsampling layer from the backbone, capturing multiscale feature information through pooling operations of different scales to enhance the model’s representation capacity. The expression for SPPF is as follows:

(7) $$Y=Conv\left(Concat\left(MaxPool\left(X,P_{1}\right),MaxPool\left(X,P_{2}\right),\cdots MaxPool\left(X,P_{i}\right)\right)\right)$$

where i represents the number of pooling windows, where each window has a size of${\;P}_{i}\times P_{i},$ and X denotes the input feature map.

**Figure 7:**
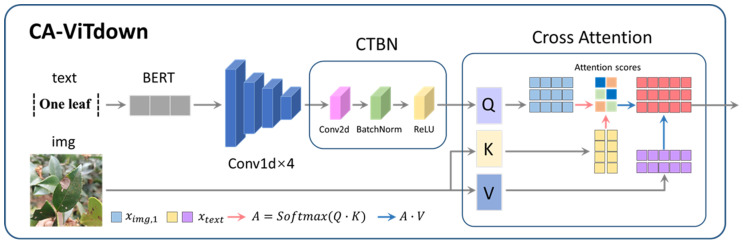
The network architecture diagrams for the DownViT and UpViT in the ViT branch on the left side and the UpCNN network structure in the CNN branch on the right side.

The multiscale fused features returned by SPPF are upsampled layer by layer to generate feature maps for image segmentation predictions. We integrate the downsampling outputs from the U-shaped CNN and the upsampling outputs from the U-shaped ViT into the PLAM, enhancing the fusion of the image and text features. These are then connected in a skip connection manner with corresponding layers in the UpCNN, thereby improving the model’s ability to distinguish between camellia diseases and similar environmental conditions. The network structures of the backbone, SPPF, and PLAM are shown in Figure 5B,C, respectively. The UpCNN network structure is shown in Figure 7.

### 4.2. ViT Branch

Similar to the U-shaped CNN branch, the U-shaped ViT branch is designed to integrate image and text features. Utilizing the BERT_12_768_12 model, each individual word is transformed into a 768-dimensional word vector [49]. Subsequently, the first layer of the CA-DownViT module receives text features from BERT-Embed and image features from the first layer of the DownCNN module. Attention weights are used to select and fuse these two types of features, enhancing the model’s capability to understand and process multimodal data.

**Figure 8:**
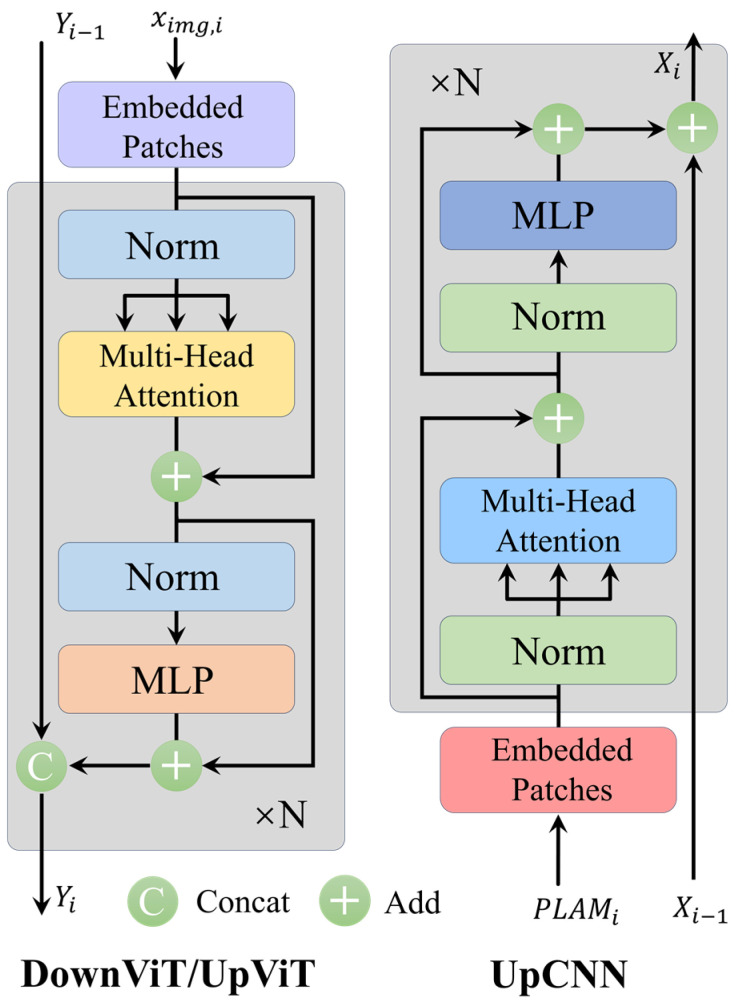
The text and image features are fused by cross-attention in CA-DownViT.

As shown in Figure 8, within the CA-DownViT module, text features are first input into a 4-layer 1D convolution to compress them, followed by the CTBN module to better align the feature dimensions of $x_{img,1}\;$and $x_{text}$. The CTBN block consists of Conv layers, BatchNorm layers, and ReLU activation layers. After separately projecting the text and image features linearly, attention scores are computed. These scores are softmax-normalized to obtain attention weights, which are then used to weigh the sum of the features. Finally, the fused output features obtained from DownViT are returned via linear projection. The specific operations for merging cross-modal features are mathematically represented by the following equations:

(8) $$CTBN=ReLU\left(BatchNorm\left(Conv2d\left(\cdot \right)\right)\right)$$

(9) $$Q=W_{Q}CTBN(x_{text}),K=W_{K}x_{img,1},V=W_{V}x_{img,1}$$

(10) $${DownViT}_{1}=V\times softmax(\frac{QK^{T}}{\sqrt{d_{k}}})$$

In this context, $x_{text}$ represents the text features; $x_{img,i}$ represents the image features from the i-th layer of the U-shaped CNN; $W_{Q}$, $W_{K},$ and$\;W_{V}$ denote the weight matrices; and $d_{k}$ is the dimensionality of the keys.

We use PatchEmbedding to form embedded features. The subsequent 2nd, 3rd, and 4th layers of the DownViT module receive features both from the preceding DownViT layer and from the corresponding CSA module. ViT employs a 2-layer Transformer encoder, passing through a Multi-Head Self-Attention (MHSA) module and a Multi-Layer Perceptron (MLP) layer sequentially, where LN represents the normalization layer, as shown in the following equations:

(11) $$x_{img,i}=PatchEmbedding\left({DownViT}_{i}\right)$$

(12) $$x_{i+1}={ViT}_{1}\left(x\right)=MHSA\left(LH\left(x\right)\right)+x$$

(13) $$Y={ViT}_{2}\left(x\right)=MLP\left(LN\left(x_{i+1}\right)\right)+x_{i+1}$$

(14) $${DownViT}_{i+1}=ViT\left({DownViT}_{i}+x_{img,i}\right)$$

The features of the corresponding dimensions are then passed through the UpViT module to the PLAM interaction module. These features are merged with those from the corresponding layers of the DownCNN module. This approach preserves low-level detail features while extracting high-level abstract features at deeper layers, mitigating the problem of gradient vanishing. It also avoids potential performance degradation caused by the inaccurate matching of textual annotations with image labels.

### 4.3. Coordinate Space Attention

In this paper, we developed a new attention mechanism, Coordinate Space Attention (CSA), based on Coordinate Attention. The Coordinate Attention introduces position encoding into the feature map to learn the relative positional relationships between each location. This allows the model to consider these relationships when calculating attention weights, thereby capturing spatial relationships between targets more effectively [50].

Coordinate Attention Coordinate Attention addresses the lack of spatial information in channel attention mechanisms like SE and the insufficient extraction of long-range relationships in mixed attention mechanisms like CBAM. It achieves this by designing a method to capture both spatial information and channel relationships [47]. Additionally, compared to non-local attention or self-attention methods, Coordinate Attention establishes long-range dependencies while reducing the computational complexity.

However, Coordinate Attention encodes only in two directions, which limits the interaction along the other direction. Therefore, CSA encodes along three directions, capturing positional relationships between each other and thereby enhancing the spatial positional information. CSA can be viewed as a computational unit that takes any intermediate feature vector X as the input and, through transformation, outputs a Y vector of the same dimensions as the X tensor while possessing enhanced representation. The network structure of the CSA is shown in Figure 6D.

GlobalAvgPooling encodes global spatial information into channel information but struggles to retain positional information [51]. To enable the attention module to better capture long-distance positional interactions, CSA decomposes global pooling into three one-dimensional encoding operations. Specifically, for a given input feature map X with dimensions H×W×C, average pooling is performed separately along the H, W, and C directions to obtain perceived feature maps $Z^{h}(H\times 1\times C)$, $Z^{w}(1\times W\times C)$, and${\;Z}^{c}(H\times W\times 1)$. This approach allows the attention module to capture long-range dependencies in each direction while preserving precise positional information, enabling the more accurate localization of important targets.

Subsequently, the obtained feature maps $Z^{h}$, $Z^{w}$, and ${\;Z}^{c}$ are transformed into the same dimensions, concatenated along the channel dimension, and then processed using a two-dimensional convolution. The specific formula is as follows:

(15) $$f=F_{1}\left(concat\left(Z^{h},Z^{w},Z^{c}\right)\right)$$

In this process, F1 represents a 2D convolution. 

The feature map f generated by this formula encodes and interacts information in three directions. The feature map f is decomposed along the channel dimension into three individual tensors: $f^{h}$, $f^{w}$, and $f^{c}$. Subsequently, the weight coefficients $g^{h}$, $g^{w}$, and $g^{c}$ for the three respective directions are

(16) $$g^{h}=\sigma \left(F_{h}\left(f^{h}\right)\right)$$

(17) $$g^{w}=\sigma \left(F_{w}\left(f^{w}\right)\right)$$

(18) $$g^{c}=\sigma \left(F_{c}\left(f^{c}\right)\right)$$

where $F^{h}$, $F^{w}$, and $F^{c}$ are 1×1 2D convolutions, and σ represents the Sigmoid activation function. Finally, $g^{h}$, $g^{w}$, and $g^{c}$ are expanded to match the dimensions of the input feature map X and are then multiplied element-wise to obtain the final output feature map Y.

CSA is a lightweight attention module designed to capture long-range dependencies, making it easily integrable into various networks. This module enhances the feature extraction capabilities while adding minimal computational overhead, thereby improving the overall performance of the network.

### 4.4. Loss Function

Focal Loss is a loss function specifically designed for multi-class tasks, particularly effective in addressing the issue of class imbalance in Camellia pest and disease categories. It modifies the cross-entropy loss by introducing a modulation factor, which allows the model to focus more on hard to classify samples during training [52]. The formula is as follows:

(19) $$FocalLoss\left(S_{t}\right)=-{\beta_{t}\left(1-S_{t}\right)}^{\gamma }\log\left(S_{t}\right)$$

In this formula, $S_{t}$ represents the predicted probability of the model for a sample, and $\beta_{t}$ is a balancing factor that adjusts the ratio between positive and negative samples. The parameter γ is a modulation factor that controls the focus on hard to classify samples. 

By incorporating the factor ${\beta_{t}\left(1-S_{t}\right)}^{\gamma }$, Focal Loss reduces the loss contribution from easy to classify samples while increasing the loss contribution from hard to classify samples, thus directing the model’s attention towards these more challenging cases.

Dice Loss is a loss function used for image segmentation tasks, measuring the similarity between two sets. It is derived from the Dice coefficient [53]. The formula is as follows:

(20) $$DiceLoss=1-\frac{2\left|A\cap B\right|}{\left|A\right|+\left|B\right|}$$

where A represents the predicted segmentation result of the model, and B represents the ground truth segmentation label. The term $\left|A\cap B\right|$ denotes the intersection area between the predicted result and the ground truth, while $\left|A\right|+\left|B\right|$ represents the sum of the sizes of the predicted result and the ground truth.

Dice Loss directly optimizes the Dice coefficient, making the model more robust in image segmentation tasks, particularly advantageous in handling small targets of Camellia diseases and pests. Since Dice Loss focuses on the overlapping area between predictions and ground truth, it naturally mitigates the class imbalance problem of plant diseases and pests, especially when dealing with the uneven distribution of Camellia disease foregrounds and backgrounds. By combining Focal Loss and Dice Loss, we leverage the strengths of both, achieving superior performance in image segmentation tasks. The combined loss function is expressed as follows:

(21) Loss=FocalLoss+DiceLoss

By combining Focal Loss and Dice Loss, we leverage their strengths in handling class imbalance and improving segmentation performance. Focal Loss focuses on hard to classify samples, enhancing the model’s performance in scenarios with class imbalance, while Dice Loss directly optimizes the segmentation metric, improving the model’s segmentation accuracy.

In addition, we propose a pixel-wise weight allocation function (PWWA) to calculate the weight of each diseased pixel type, matching and optimizing the loss function during training. First, the model converts the disease class colors to grayscale values and counts the number of pixels and images for each grayscale value. Then, it calculates the frequency of pixels of a specific disease class color across all images. Finally, CTDUNet determines the median of these frequencies and the ratio of each color frequency to the median frequency, helping to understand the degree of deviation of each color frequency from the median. The formula is as follows:

(22) $${Fre}_{i}=\frac{pixs}{n_{i}\times w\times h}$$

(23) $$Fre=\left[{Fre}_{0},{Fre}_{1},\ldots \ldots {Fre}_{i-1},{Fre}_{i}\right]$$

(24) $${Fre}_{median}=Median\left(array\left(Fre\right)\right)$$

(25) $$F_{i}=\frac{{Fre}_{median}}{{Fre}_{i}}$$

where pixs represents the frequency of each diseased pixel type, $n_{i}$ is the number of images containing the i disease type pixels, and w and h represent the width and height of the image, respectively. $F_{i}$ denotes the degree of deviation of the frequency of each disease type from the median frequency.

## 5. Conclusions

This paper introduces a CNN–Transformer-based dual U-shaped visual–language multimodal model (CTDUNet) that leverages textual information to compensate for deficiencies in image data quality. We evaluated the performance of the CTDUNet model on a self-constructed dataset of *Camellia oleifera* pests and diseases. The experimental results indicate that our model achieves 86.14% mIoU on images with a resolution of just 224 × 224, demonstrating its excellent segmentation performance in complex environmental backgrounds and validating the effectiveness of textual information. Additionally, it demonstrated excellent sample balance, effectively addressing the previously mentioned challenges.

We designed a dual U-shaped network structure to deeply extract and integrate image and textual information, fully utilizing the powerful cross-modal processing capabilities of Transformers. This strengthens the model’s ability to handle the complexities of *Camellia oleifera* pests and diseases. To address the challenges of overlapping diseased leaves and multi-disease leaves, we proposed the CSA mechanism, which innovatively focuses on features from the C, H, and W dimensions. This helps the model accurately locate important targets, concentrate on fine details, and effectively distinguish overlapping leaves. CTDUNet also employs the cross-attention mechanism (CA) to cross-fuse textual and image features, ensuring alignment between the image and textual features. The fused features, along with the image features, are then input into the PLAM. The PLAM merges the corresponding channel features with similar semantics, preserving as much local image feature information as possible.

In this study, we only performed a simple vectorization of the text. We will adopt more complex, yet lightweight, text encoding methods to capture richer semantic information and contextual relationships in the text. Additionally, we will introduce a dynamic feature extraction mechanism that enhances the fusion of image and textual data through an adaptive attention mechanism, thereby improving the model’s flexibility and adaptability. 

## Figures and Tables

**Table 1:** Comparison of a published dataset of *Camellia oleifera* pests and diseases with a homemade dataset. The symbol “×” indicates the absence of the characteristic, while “√” indicates its presence.

Dataset	Category	Amount	Complex Background	Text	Mask
Image recognition of *Camellia oleifera* diseases based on convolutional neural network & transfer learning [Long Mansheng]	4	3750	×	×	×
Classification of *Camellia oleifera* Diseases in Complex Environments by Attention and Multi-Dimensional Feature Fusion Neural Network [Yixin Chen]	7	12,170	√	×	×
Ours	7	1400	√	√	√

**Table 2:** Parameters of the device and environment.

Environment	Device	Parameter
Hardware environment	CPU	AMD EPYC 7763
	GPU	NVIDIA RTX 4090
	RAM	64 G
	Video memory	24 G
Software environment	OS	Ubantu 22.04
	CUDA Toolkit	11.5
	CUDNN	8.9.7
	Python	3.7.12
	Pytorch-GPU	1.8.0
	torchvision	0.9.0

**Table 3:** Setting of the experiment.

Hyperparameters	Parameters
Size of input images	224 × 224
Batch	16
Initial learning rete	0.0001
Optimizer	Adam
Momentum	0.9

**Table 4:** Comparison of the evaluation of *Camellia oleifera* disease segmentation models. The best case scenarios for each indicator are highlighted in bold. In this context, ‘×’ denotes the absence of textual feature input, while ‘√’ indicates the presence of textual feature input.

Model	Text	Precision (%)	Recall (%)	Dice (%)	mIoU (%)
DeeplabV3+-mobilnet	×	85.91	87.36	86.63	77.12
DeeplabV3+-xception	×	83.63	87.25	85.40	75.33
Segformer-b2	×	90.18	89.13	89.65	81.76
Segformer-b5	×	92.45	87.73	90.03	82.21
PSPNet-MobilenetV2	×	79.56	70.61	74.82	61.73
PSPNet-Resnet50	×	83.96	74.82	79.13	67.22
HrNetV2-W18	×	86.59	83.87	85.20	74.96
HrNetV2-W32	×	89.51	85.93	87.68	78.66
HrNetV2-W48	×	89.97	87.03	88.48	80.01
UNet-Vgg	×	86.68	89.15	87.90	78.64
UNet-Resnet	×	83.42	86.76	85.01	74.66
LViT	√	**95.28**	85.50	89.26	80.73
**CTDUNet (Ours)**	√	93.69	**91.24**	**92.45**	**86.14**

**Table 5:** Comparison of the evaluation of attention mechanisms. The best case scenarios for each indicator are highlighted in bold.

Attention (CTDUNet)	Precision (%)	Recall (%)	Dice (%)	mIoU (%)
SE [44]	92.63	90.49	91.55	84.60
CBAM [45]	93.10	90.44	91.75	85.00
EMA [46]	93.16	90.73	92.18	85.30
Coordinate Attention [47]	93.67	90.73	92.18	85.67
Coordinate Space Attention (Ours)	**93.69**	**91.24**	**92.45**	**86.14**

**Table 6:** Comparison of the CTDUNet ablation experiments by module. The best case scenarios for each module are highlighted in bold. In this context, ‘×’ denotes the absence of textual feature input, while ‘√’ indicates the presence of textual feature input.

Method	Text	Precision (%)	Recall (%)	Dice (%)	mIoU (%)
LViT (base)	√	91.91	86.38	89.06	80.27
CTDUNet (base)	√	92.85	89.30	91.04	83.63
CTDUNet (base)	×	90.51	86.97	88.70	79.83
CTDUNet + (SPPF + CA-DownViT)	√	93.08	90.42	91.73	84.93
CTDUNet + CSA	√	93.00	90.38	91.67	84.81
CTDUNet + (SPPF + CA-DownViT) + CSA	×	91.11	87.49	89.26	80.73
**CTDUNet + (SPPF + CA-DownViT) + CSA**	√	**93.69**	**91.24**	**92.45**	**86.14**

**Table 7:** Comparison of IoU for nine classes between CTDUNet and the other models. The best case scenarios for each indicator are highlighted in bold.

Model (IoU%)	Background	Leaf	Tea White Scab	Worm Holes	Red Leaf Spot	Algae Leaf Spot	Tea Sooty Mold	Soft Rot	Anthracnose
DeeplabV3+	95.88	92.39	62.38	64.54	86.27	73.42	79.37	84.95	67.91
Segformer	**96.85**	**94.05**	64.92	69.27	**87.79**	83.22	84.04	86.33	73.41
PSPNet	94.95	89.66	16.99	49.63	79.54	65.72	69.84	80.03	58.67
HrNetV2	95.76	92.3	59.47	66.64	86.21	77.32	79.85	88.76	73.78
UNet	92.8	89.37	68.09	67.8	82.68	75.66	75.66	85.68	69.75
LViT	93.25	89.03	69.58	70.63	82.39	78.79	85.6	85.46	67.78
CTDUNet	95.23	91.98	**75.29**	**78.96**	86.15	**85.49**	**87.91**	**94.67**	**79.57**

## Data Availability

Requests to access the datasets should be sent via email to guo1750524639@163.com.
